# Modeling Pathways to Describe How Maternal Health Care Providers' Mental Health Influences the Provision of Respectful Maternity Care in Malawi

**DOI:** 10.9745/GHSP-D-23-00008

**Published:** 2023-11-30

**Authors:** Brady Burnett-Zieman, Charlotte E. Warren, Felistas Chiundira, Edina Mandala, Fannie Kachale, Christina Heather Mchoma, Alexander Mboma, Martha Kamanga, Abigail Kazembe

**Affiliations:** a Population Council, Washington, DC, USA.; b Kamuzu University of Health Sciences, Lilongwe, Malawi.; c Reproductive Health Directorate, Ministry of Health, Lilongwe, Malawi.; d Kamuzu University of Health Sciences, Lilongwe, Malawi.

## Abstract

**Introduction:**

Maternal health (MH) providers may experience traumatic events, such as maternal or fetal death, that can contribute to stress and burnout. Past trauma, poor working relationships, and under-resourced environments not only affect providers' own emotional well-being but also reduce their ability to provide respectful maternity care (RMC).

**Methods:**

Data were collected in mid-2021 as a cross-sectional survey with 302 MH providers working in 25 maternities in 3 districts in Malawi to measure burnout, depression, and post-traumatic stress disorder (PTSD). We present a pathway model describing how these factors interact and influence RMC. We used the provider-reported person-centered maternity care scale to measure RMC; the Maslach Burnout Inventory, which examines emotional exhaustion, depersonalization, and professional accomplishment; and standard validated screening tools to measure the prevalence of depression and PTSD.

**Results:**

Although levels of burnout varied, 30% of MH providers reported high levels of exhaustion, feelings of cynicism manifesting as depersonalization toward their clients (17%), and low levels of professional accomplishment (42%). Moderate to severe depression (9%) and suicidal ideation within the past 2 weeks (10%) were also recorded. Many (70%) reported experiencing an event that could trigger PTSD, and 12% reported at least 4 of 5 symptoms in the PTSD scale. Path analysis suggests that depression and emotional exhaustion negatively influence RMC, and depersonalization is mediated through depression. PTSD has no direct effect on RMC, but increased PTSD scores were associated with increased burnout and depression scores. Positive relationships with facility managers were significantly associated with increased RMC and decreased emotional exhaustion and depersonalization.

**Conclusion:**

Burnout will continue to be a challenge among MH providers. However, pragmatic approaches for improving teamwork, psychosocial, and managerial support for MH providers working in challenging environments may help mitigate burnout, improve MH provider well-being, and, in turn, RMC for women seeking MH services.

## Abstract (precis)

Measuring provider burnout and understanding how it impacts delivery of maternity care can help address ways to improve respectful care. Improving facility management is essential to mitigate provider depression, emotional exhaustion & burnout.

## Teaser

**Key Messages:**

- Without identifying and addressing burnout among maternal health (MH) providers, efforts to achieve respectful maternity care (RMC) will not be reached. Providers report being emotionally exhausted and depersonalizing their clients, and one-tenth experience depression, post-traumatic stress disorder (PTSD), or suicidal ideation.
- We found correlations between mental health factors and RMC. A path analysis indicates that emotional exhaustion and depression appear to directly influence RMC; PTSD and depression have secondary effects, which are mediated through exhaustion and depression.
- The path analysis also shows that having positive, supportive working relationships with facility managers is strongly associated with improved RMC. Moreover, positive working relationships may also protect MH providers against some of the more pernicious effects of burnout.

**Key Implication:**

- Addressing burnout is critical to improving RMC in Malawi and elsewhere. Policymakers and health managers should recognize how burnout is shaped by providers' day-to-day experiences, as well as coincident mental health conditions, including PTSD and depression.

## INTRODUCTION

To optimize the performance, quality, and impact of a maternal health workforce, it is essential to understand the experiences of health care providers and challenges they may face. Maternal health (MH) providers often experience traumatic events, such as maternal or fetal death, that can cause them sadness and grief.^1^ Other critical incidents that are less serious than death can be mentally draining and disturb a provider's sense of peace and purpose. Poor mental health, including poor working relationships and under-resourced environments, not only affects MH providers' emotional health but also affects how they treat women during childbirth.^2^ Poor MH provider attitude is often cited as a reason why women may not seek care or delay seeking care at facilities. Over the last decade, this issue has gained increased focus, including how providers may at times fail to provide respectful maternity care (RMC), leading to poor birth outcomes.^3^^,^^4^

One of the documented driving factors that contribute to a lack of RMC is the challenge of providers working in a weak health system that is exacerbated by inadequate infrastructure (beds, space, and utilities), lack of appropriate equipment, medicines, and supplies, and an insufficient health workforce.^4^^–^^6^ Even when MH providers are present, many are unable to adequately attend births and provide quality care due to high client volume and/or staffing shortages. Delays in admission and incorrect assessment and treatment are contributing factors in many facility-based deaths.^7^ Midwifery and nursing staff are frequently reassigned or rotated to new wards or facilities, often replacing more experienced MH providers with new recruits or otherwise inexperienced staff.

Because of unpredictable client loads, understaffing, inexperience, and poor management, burnout among MH providers is common. Many consider labor and delivery unit assignments to be among the most stressful jobs in a health facility.^1^^,^^8^ This issue can be compounded by facility culture. When supervisors are not sufficiently accountable to their staff, MH providers do not receive essential skills updates and job support, lack the confidence to manage obstetric complications, and may fail to consistently follow standard procedures and guidelines.^6^^,^^9^^,^^10^

Because of unpredictable client loads, understaffing, inexperience, and poor management, burnout among maternity care providers is common.

Burnout is a syndrome that develops in response to a health care provider's daily experiences and can negatively impact provider motivation and the delivery of health services. Occurring at the confluence of economic, professional, and social barriers, burnout affects health care providers' well-being and motivation and can lead to low-quality care.^8^^,^^11^ During a cocreation process in 2019 as part of the Advancing Postpartum Hemorrhage Care partnership, MH stakeholders identified factors such as MH provider motivation, psychosocial stressors, facility culture, and others that contribute to burnout as critical barriers to providing quality emergency obstetric care in Malawi. Nevertheless, burnout is overlooked in health care provider behavior change programming, including in programs that focus on improving the quality of RMC. Prior research suggests that burnout may compound performance issues, such as inadequate adherence to standard procedures,^12^ and subsequently exacerbate the mistreatment of women, failure to detect and treat complications, and ultimately increase maternal morbidity and mortality.^4^^,^^13^

The Maslach Burnout Inventory (MBI) was originally developed in 1986 and has since become a standard measure of burnout among health care professionals. The MBI has undergone extensive validation and has been cited in more than 1,000 studies across high, middle- and low-income countries and with a wide array of health care provider cadres.^14^ The MBI comprises 3 dimensions: emotional exhaustion, depersonalization, and professional accomplishment.^14^

As measured by the MBI, burnout is characterized by high levels of emotional exhaustion, depersonalized attitudes toward clients, and low professional accomplishment. A systematic review published in 2018 indicates that health care providers throughout low- and middle-income countries throughout Asia, Europe, Latin America, Mideast/North Africa, and sub-Saharan Africa experience 1 or more of these aspects of burnout on a regular basis.^15^ In Malawi, studies using the MBI have shown that burnout is particularly prevalent among maternal health staff who experienced burnout more frequently than colleagues working in other medical settings.^8^ Burnout was also found to be associated with self-reported suboptimal care provision and attitudes among HIV service providers.^12^ Studies using the MBI in other countries have shown associations between burnout and staff turnover or intention to quit,^16^ suicide,^17^ a reduction in perceived quality of service delivery and objective client safety.^12^^,^^18^^,^^19^

In addition to burnout, depression and PTSD are notably prevalent in health care providers worldwide, especially in resource-limited settings.^20^ Burnout, typified by emotional exhaustion and depersonalization, often co-occurs with PTSD and depression, forming a triad that significantly impacts health care delivery and the lives of health care workers.^21^^–^^23^

In low-resource regions like sub-Saharan Africa, the occurrence of PTSD and depression among health care providers is concerning.^24^ These mental health disorders can be driven by the high-stress nature of their work, insufficient staffing or supplies,^25^ and exposure to traumatic events,^26^ common in areas with high maternal mortality rates. The co-occurrence of these disorders with burnout further exacerbates the challenges, leading to reduced personal accomplishment, decreased productivity, and suboptimal client care.^12^

To form a more holistic picture of mental health status among Malawian MH providers and develop a better understanding of how these mental health issues are interrelated, we included 2 additional measures: the Patient Health Questionnaire-9 (PHQ-9),^27^ a 9-item screening tool for major depression, and the 5-item Primary Care PTSD Screen for DSM-5 (PC-PTSD-5).^28^

This article seeks to expand the literature on factors related to MH provider burnout and self-reported RMC to improve understanding of barriers to providing quality obstetric care. Our objectives were to (1) measure depression, PTSD, emotional exhaustion, depersonalization, and personal accomplishment among providers working in maternity units in Malawi; (2) assess MH provider self-reported levels of RMC; and (3) develop a pathway model to help examine the interaction of factors that affect MH provider mental health and RMC.

## METHODS

### Study Context

This study is part of a larger implementation research portfolio supported by the U.S. Agency for International Development through the Advancing Postpartum Hemorrhage Care partnership in Malawi. The partnership includes the Population Council's Breakthrough RESEARCH project and the HEARD Project at University Research Co., as well as Kamuzu University of Health Sciences and Reproductive Health Directorate at the Ministry of Health (MOH) in Malawi. Representatives of the Malawi Safe Motherhood Technical Working Group, chaired by the MOH, convened with other MH stakeholders to explicate contextually relevant research priorities on improving prevention, early detection, and management of postpartum hemorrhage across different facility levels and districts. Although several other studies have reported the co-occurrence of health care provider burnout, depression, and PTSD during the COVID-19 pandemic, this article may be the first to present data collected in sub-Saharan Africa or from MH providers.

### Study Setting and Data Collection

We used data from a cross-sectional survey with MH providers working in maternity units in Malawi. After consultation with the MOH and a bilateral maternal and newborn health care and health systems strengthening activity supported by the U.S. Agency for International Development, 25 health facilities were purposively selected from 4 districts in central and southern Malawi (Dowa, Balaka, Lilongwe, and Zomba). Each facility conducted at least 25 deliveries per month and included 11 primary health care centers, 11 community or rural hospitals, and 3 district or referral hospitals.

Between April and May 2021, data were collected from 302 MH providers from the 25 selected facilities. Due to the small number of MH providers available and high turnover, the research team attempted to interview all eligible MH providers in the targeted facilities. Eligible MH providers were physicians, obstetricians, clinical officers, nurses, midwives, and nurse midwife technicians who worked in or supervised the maternity unit of 1 of the 25 study facilities.

All participants were aged at least 18 years and provided verbal consent. Research staff obtained lists of MH providers from facility administrators and contacted the providers by phone, invited them to participate, obtained informed consent, and coordinated a convenient time and private place to complete the survey.

Because data collection occurred during the early phases of the COVID-19 pandemic, all surveys were conducted by telephone to limit the risk of COVID-19 transmission. Each survey lasted 40–50 minutes and was conducted in English or Chichewa. The Midwifery Department at Kamuzu University of Health Sciences coordinated the data collection team and set up a call center in Lilongwe to complete all provider surveys; comprehensive COVID-19 prevention procedures were used to avoid disease transmission between data collectors at the call center.

During the phone-based surveys, the interviewer entered MH providers' responses directly into electronic survey forms. Questions included not only providers' direct knowledge about how to properly manage an obstetric emergency, including postpartum hemorrhage, but also self-reported experience of providing RMC and behavioral factors that could influence their ability to respond to an obstetric emergency, such as MH providers' experiences interacting with supervisors or facility management, stressors and burnout, traumatic events, PTSD, and depression.

### Measures

The primary outcome of interest in this analysis is MH providers' self-reported provision of RMC. To measure RMC, we used the 9-question provider-reported person-centered maternity care (PCMC) scale.^29^ Each question uses a 4-part Likert response set—ranging from “no, never” to “all the time”—to examine a specific behavior related to the delivery of RMC. Supplement 1 provides the full set of questions.^28^

This recently developed measure is derived from the well-established client-reported 30-item PCMC scale, in which scores indicating more respectful care have been associated with reduced rates of maternal depression, as well as maternal and newborn complications, which has shown associations. In 2021, Afulani et al. published research describing the validation of this instrument in Kenya and Ghana. Factor analysis results from this study indicate a unidimensional factor structure; all 9 items had acceptable loading scores (>0.3) and internal reliability (Cronbach's α=0.72).^30^ The distribution of responses collected using the provider-reported PCMC scale was generally consistent with clients' responses to corresponding questions in the PCMC scale.^30^ For clarity, we will continue to use RMC when discussing PCMC scores, as RMC is the construct measured by this scale.

To assess MH provider well-being and mental health, we used 3 different measures: the MBI, depression scales, and PTSD scales. The MBI has a 3-factor structure comprising: emotional exhaustion (9 items, range: 0–54), depersonalization (5 items, range: 0–30), and professional accomplishment (8 items, range: 0–48). Each item in the MBI uses a 7-part set of Likert-scaled response options ranging from “never (0)” to “every day (6)”(Supplement 2). Since its creation in 1986, the MBI has been used to measure burnout among health care providers around the world. Initial validation studies indicate acceptable (α>0.7) for all factors; however, some studies have indicated lower reliability of depersonalization. Test-retest reliability was lowest for depersonalization and highest for emotional exhaustion.

To create the initial descriptive tables, we used the methods recommended by Maslach and Leitner to set population-standardized thresholds for each of the 3 subdomains^14^: (1) an emotional exhaustion score of 22 or higher indicates high levels of exhaustion; (2) a depersonalization score of 10 or higher indicates high levels of cynicism, and (3) a professional accomplishment score lower than 38 indicates low levels of professional accomplishment.

To examine the co-occurrence of depression and PTSD among MH providers, we included the PHQ-9 depression screening tool and the PC-PTSD-5 screening tool. The PHQ-9 comprises 9 questions that measure the frequency that respondents have experienced key features of depression during the preceding 2 weeks (Supplement 3). The PHQ-9 has been extensively validated in various populations,^27^ including sub-Saharan Africa. In a study conducted in Nigeria, the PHQ-9 demonstrated good internal consistency with a Cronbach's alpha coefficient of 0.85, good test-retest reliability (r=0.89), and high sensitivity (0.85) and specificity (0.994) in detecting severe depression using a cutoff score of 10.^31^ Each question uses a 4-part set of Likert-scaled responses, ranging from “not at all (0)” to “nearly every day (3).” A total score of 10 or higher is indicative of moderate to severe depression or depression that warrants a treatment plan that may include medication.

The PC-PTSD-5 (Supplement 4) is a 6-item screening tool designed to identify individuals with PTSD. The first item establishes whether the respondent has previously had exposure to traumatic events. The remainder of the tool comprises 5 yes/no questions that assess how the traumatic events have affected the respondent during the past month. Prior validation work indicates that this screening tool has excellent diagnostic accuracy. One study showed that a cutoff of 4 provides exceptional sensitivity (100%) while preserving acceptable specificity (85%).^32^ In some settings, a score of 3 or higher may be indicative of PTSD.^28^

Finally, to examine facility culture, influence of MH provider behavior, and the relationships between providers and their managers, we used a 15-item bank of validated questions. Questions include manager treats me with kindness and consideration, makes job decisions in an unbiased manner, treats me with dignity and respect, discusses implications of decisions with me, allows staff to appeal or challenge job decisions, deals with me in a truthful manner, frequency of supervision, opportunities for attending training updates and promotion.^33^

### Path Analysis

The purpose of this exploratory analysis was to propose a model that shows how multiple interconnected mental health factors influence a provider's delivery of person-centered, respectful care in maternity settings. We began by generating basic descriptive statistics for each of the 7 measures examined, after which we used Cronbach's alpha to assess internal consistency of each validated scale among this sample of MH providers, setting 0.70 as the threshold for reliability. Next, we computed a correlation matrix to assess bivariate relationships between our measures and produced 2 series of scatterplots to visualize the relationships between each measure of mental health and well-being and the outcome: RMC, as measured by the MH provider self-reported PCMC scale.

The final stage of our analysis was the iterative development and refinement of a path diagram to describe relationships between emotional exhaustion, depersonalization, professional accomplishment, depression, PTSD, the management inventory, and the provision of RMC. Path analysis is a tool used to understand potential causal pathways between explanatory variables and an outcome using correlational data.^34^ The path diagram is a depiction of those relationships. To this end, we specified PTSD and management inventory scores as exogenous variables in the path diagram, as both are predicated on specific experiences independent of the MH provider's self-perceived mental health status or ability to deliver care. PTSD originates from prior experience of a traumatic event or series of traumatic events; the management inventory scores are dependent on the MH provider's interactions with their supervisors and facility management structures.

Conversely, emotional exhaustion, depersonalization, and depression were specified as intermediate endogenous variables, as they represent an MH provider's internal and subjective experience of a range of unmeasured external factors. We specified a unidirectional path from the exogenous PTSD and management inventory variables to the RMC outcome variable, specifying both direct relationships between these variables and pathways between these variables that operate through the intermediate MBI-EE, MBI-DP, and depression variables. As the emotional exhaustion and depersonalization inventories were developed together to measure co-occurring aspects of burnout, we permitted the correlation of error terms between these variables in the final path diagram.

After initial model specification, we iteratively refined connections between variables, pruning those that did not add significant explanatory power to the path diagram or those that significantly reduced model fit. Goodness-of-fit for the final model was tested using the likelihood ratio (*P*>.05), standardized root mean square residual (<0.8), root mean square of error approximation (<0.05), comparative fit index (>0.9), and Tucker-Lewis Index (>0.9). Professional achievement showed minimal direct or indirect effects on RMC and was ultimately excluded from the final path model. All analyses were completed using Stata 14.0.

### Ethical Approval

Ethics approval was obtained from Population Council's Institutional Review Board #910 and College of Medicine Research and Ethics Committee, Kamuzu University of Health Sciences, Malawi #2822.

## RESULTS

### MH Provider Characteristics

The characteristics of the MH providers are presented in Table 1. Just more than half of providers (52%) worked in rural or community hospitals; one-quarter worked in primary health care centers, and the remaining 23% worked at 3 district or referral-level facilities. MH providers interviewed were nurse-midwife technicians (59%), registered nurses or midwives (24%), physicians or clinicians (10%), and community midwife assistants (7%). While the majority of study participants were female (70%), males were over-represented among physicians and clinicians, comprising nearly two-thirds (65%) of this cadre. Only 26% of registered nurses and midwives, 28% nurse/midwife technicians, and 10% of community midwife assistants were male (data not shown).

**TABLE 1.** Characteristics of Maternal Health Providers From 25 Health Facilities in Malawi

	No. (%)
Age, years	
20–29	150 (49.7)
30–39	112 (37.1)
40–49	35 (11.6)
50+	5 (1.7)
Sex	
Woman	210 (69.5)
Man	92 (30.5)
Provider cadre	
Physician/clinician	31 (10.3)
Registered nurse/midwife	72 (23.8)
Nurse/midwife technician	179 (59.3)
Community midwife assistant^a^	20 (6.6)
Facility type	
Primary health care center	76 (25.2)
Rural/community hospital	157 (52.0)
District/referral hospital	39 (22.8)
Number of years as a health professional	
<1 year	3 (1.0)
1–2 years	65 (21.5)
3–5 years	135 (44.7)
6+ years	99 (32.8)
Work locations^b,c^	
Maternity admission	103 (34.1)
Antenatal ward	65 (21.5)
Postnatal ward	101 (33.4)
Labor and delivery	217 (71.9)
Nursery/newborn unit/KMC unit	38 (12.5)
Mental health status^b^	
High exhaustion (EE: 22 or higher)	91 (30.1)
High cynicism (DP: 10 or higher)	50 (16.6)
Low professional accomplishment	127 (42.1)
Moderate to severe depression	24 (8.0)
Post-traumatic stress disorder	36 (11.9)

Abbreviations: DP, depersonalization; EE, emotional exhaustion; KMC, kangaroo mother care; MH, maternal health.

a Community midwife assistant is a certificate-level role requiring 18 months post-secondary school training.

b Total exceeds 100%.

c May work in more than 1 location.

Approximately half of MH providers interviewed were aged 20–29 years, and over three-quarters (78%) had at least 3 years of experience as a provider. Participants reported working across multiple departments within the maternity unit. Nearly three-quarters of MH providers said they worked in labor and delivery; one-third said they worked in the postnatal ward and/or maternity admissions. Fewer MH providers worked in the antenatal ward (22%) or cared for newborns (13%). Table 1 also describes providers self-reporting on their current mental health status. Around 30% of MH providers reported being exhausted, 17% reported depersonalizing or feeling cynical toward their clients, and 42% reported low levels of professional accomplishment.

Around 30% of MH providers reported being exhausted, 15% reported feeling cynical, and 42% reported low levels of professional accomplishment.

The Cronbach's alpha for the emotional exhaustion, depersonalization, professional accomplishment, and PHQ-9 scales were α=0.711, α=0.588, α=0.727, and α=0.828, respectively (Table 2). Scores on the PHQ-9 depression scale of 10 or higher are indicative of moderate to severe depression or depression that would warrant medication as part of a comprehensive treatment plan. While nearly 9% of participants had scores consistent with moderate to severe depression, 60% of the 165 participants who reported any depressive symptoms said that their symptoms made their work at least somewhat more difficult (data not shown). And 10% reported suicidal ideation within the past 2 weeks.

**TABLE 2.** Mental Health Measures Among Maternal Health Providers in Malawi

	N	Items	Maximum Score	Range	Mean	SD	Cutoff	Alpha
MBI–emotional exhaustion	302	9	54	0–48	17.1	9.6	22 or higher	0.711
MBI–depersonalization (cynicism)	302	5	30	0–22	4.3	4.5	10 or higher	0.588
MBI–professional accomplishment	302	8	48	8–48	38.1	8.6	Less than 39	0.727
PHQ-9 depression scale	302	9	27	0–20	3.1	4.1	10 or higher	0.828
PTSD screening tool	302	5	5	0–5	1.2	1.5	4 or higher	0.678
Management inventory	302	13	52	3–52	31.6	8.8	N/A	0.915
Perceptions of respectful care	302	15	45	19–45	32.2	5.4	N/A	0.779

Abbreviations: MBI, Maslach Burnout Inventory; N/A, not applicable; PTSD, post-traumatic stress disorder; PHQ, Patient Health Questionnaire; RMC, respectful maternity care; SD, standard deviation.

Most participants (70%) reported experiencing an event that could trigger PTSD, and 12% reported experiencing at least 4 of the 5 PTSD symptoms listed in the PC-PTSD-5 screening questionnaire. The alpha for the 5-item assessment (α=0.678) is adequate but below the ideal threshold of 0.7. Lower internal reliability scores for both depersonalization and PTSD are consistent with prior research^8^^,^^12^ and suggest that these measures may function differently in Malawi.

### Associations Between Mental Health and RMC

**FIGURE 1:**
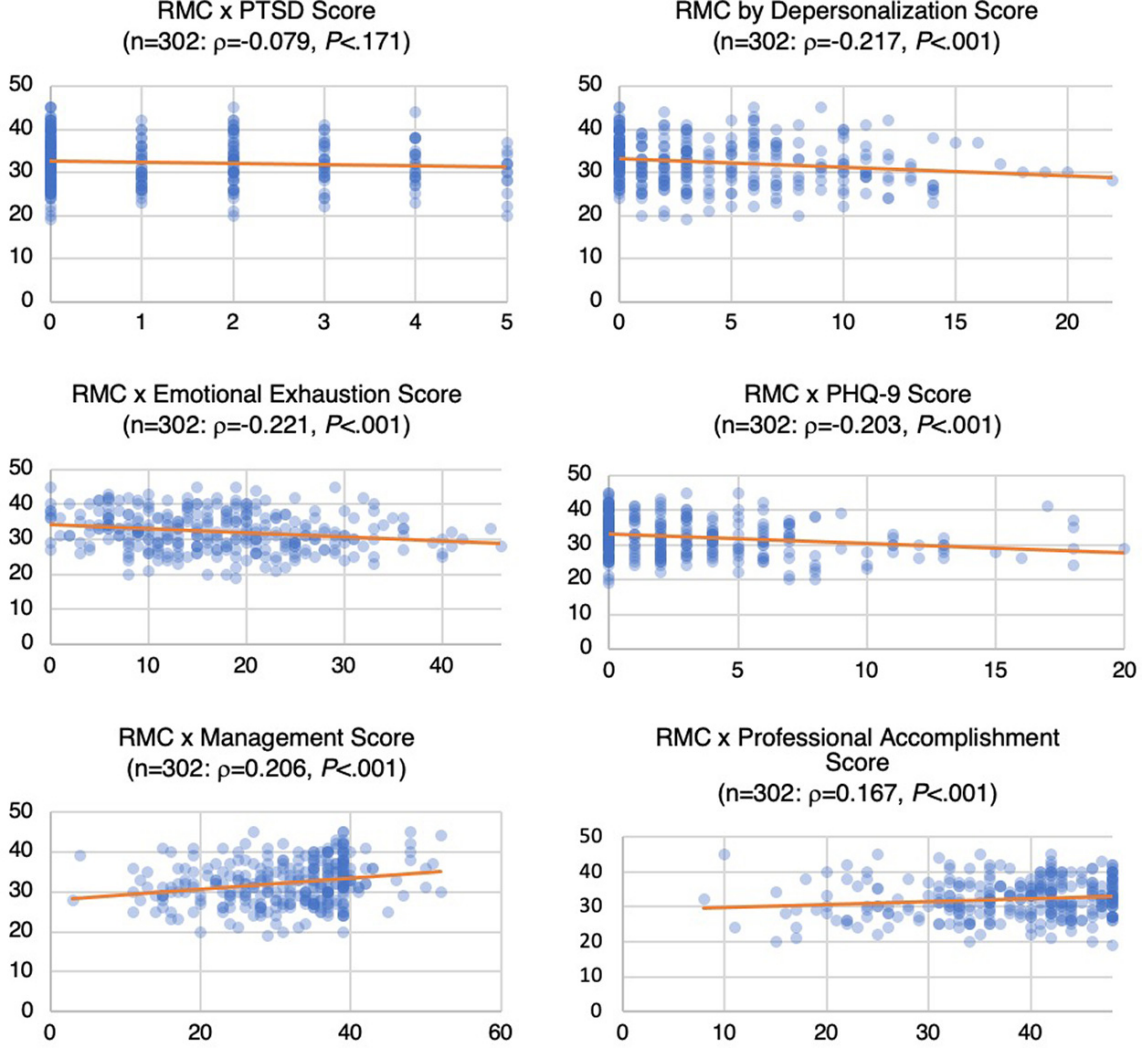
Associations Between Maternal Health Provider-Reported RMC Scores and Other Mental Health Factors, Malawi Abbreviations: PHQ-9, Patient Health Questionnaire-9; PTSD, post-traumatic stress disorder; RMC, respectful maternity care.

Figure 1 shows the relationship between each measure and MH provider-reported RMC scores. Depersonalization, emotional exhaustion, and depression scores all showed significant inverse correlations with RMC, suggesting that depression, exhaustion, and depersonalization may have a negative influence on the provision of RMC. By comparison, these data suggest that as scores for both the management inventory and professional accomplishment rose, so did RMC scores. Both factors exhibited a significant positive correlation with RMC scores. However, PTSD did not appear to be directly associated with RMC scores.

**FIGURE 2:**
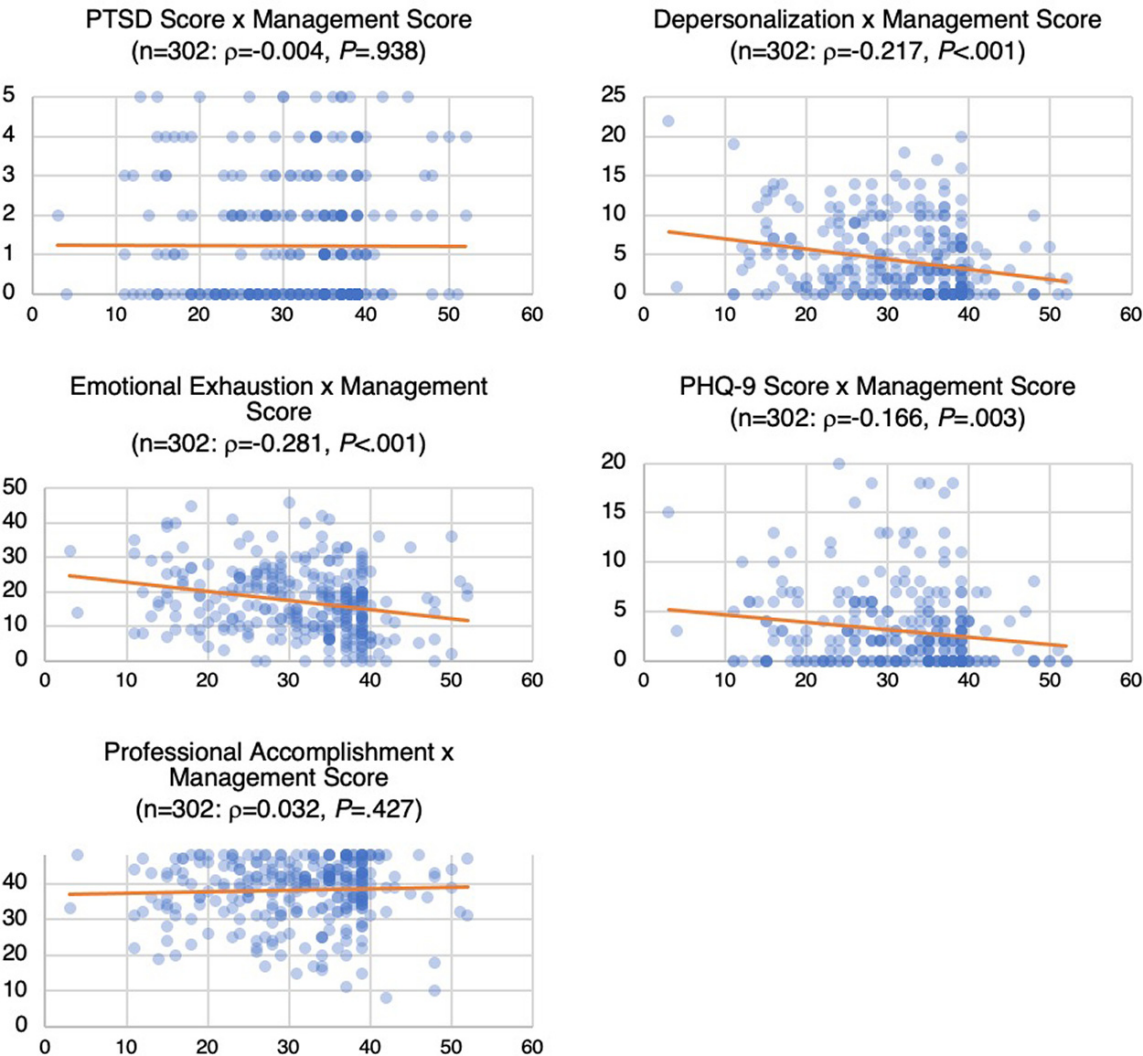
Associations Between Management Inventory Scores and Other Mental Health Factors, Malawi Abbreviations: PHQ-9, Patient Health Questionnaire-9; PTSD, post-traumatic stress disorder.

Figure 2 depicts associations between mental health factors and management inventory scores. Here, we examine how MH providers' mental health scores changed as management inventory scores increase. As seen with RMC scores, MBI-DP, MBI-EE, and depression all show a significant inverse relationship with management inventory scores: as management inventory scores increased, MBI-DP, MBI-EE, and depression scores all decreased. However, neither PTSD nor professional accomplishment scores were significantly correlated with management inventory scores.

Table 3 shows significant intercorrelation among nearly all measures captured in this survey. Although only PTSD scores were not significantly correlated with RMC, PTSD scores were correlated with emotional exhaustion (ρ=0.167, *P*=.003), MBI-DP (ρ=0.167, *P*=.003), and depression (ρ=0.335, *P*<.001), suggesting that these factors may act as mediators for the effects of PTSD on RMC.

**TABLE 3.** Correlation Matrix Between Mental Health Measures and RMC

	MBI-EE	MBI-DP	MBI-PA	PHQ-9 Depression	PC-PTSD-5	Management Inventory	RMC
MBI-EE	1						
MBI-DP	0.530	1					
MBI-PA	−0.009	−0.162	1				
PHQ-9 depression	0.268	0.414	−0.157	1			
PC-PTSD-5	0.167	0.167	−0.091	0.335	1		
Management inventory	−0.281	−0.217	0.033	−0.166	−0.0045	1	
RMC	−0.221	−0.217	0.167	−0.203	−0.0789	0.206	1

Abbreviations: DP, depersonalization; EE, emotional exhaustion; MBI, Maslach Burnout Inventory; PA, professional accomplishment; PC-PTSD-5, primary care post-traumatic stress disorder screen for DSM-5; PHQ, Patient Health Questionnaire; RMC, respectful maternity care.

### Path Analysis: Associations Between Mental Health, Protective Factors, and RMC

**FIGURE 3:**
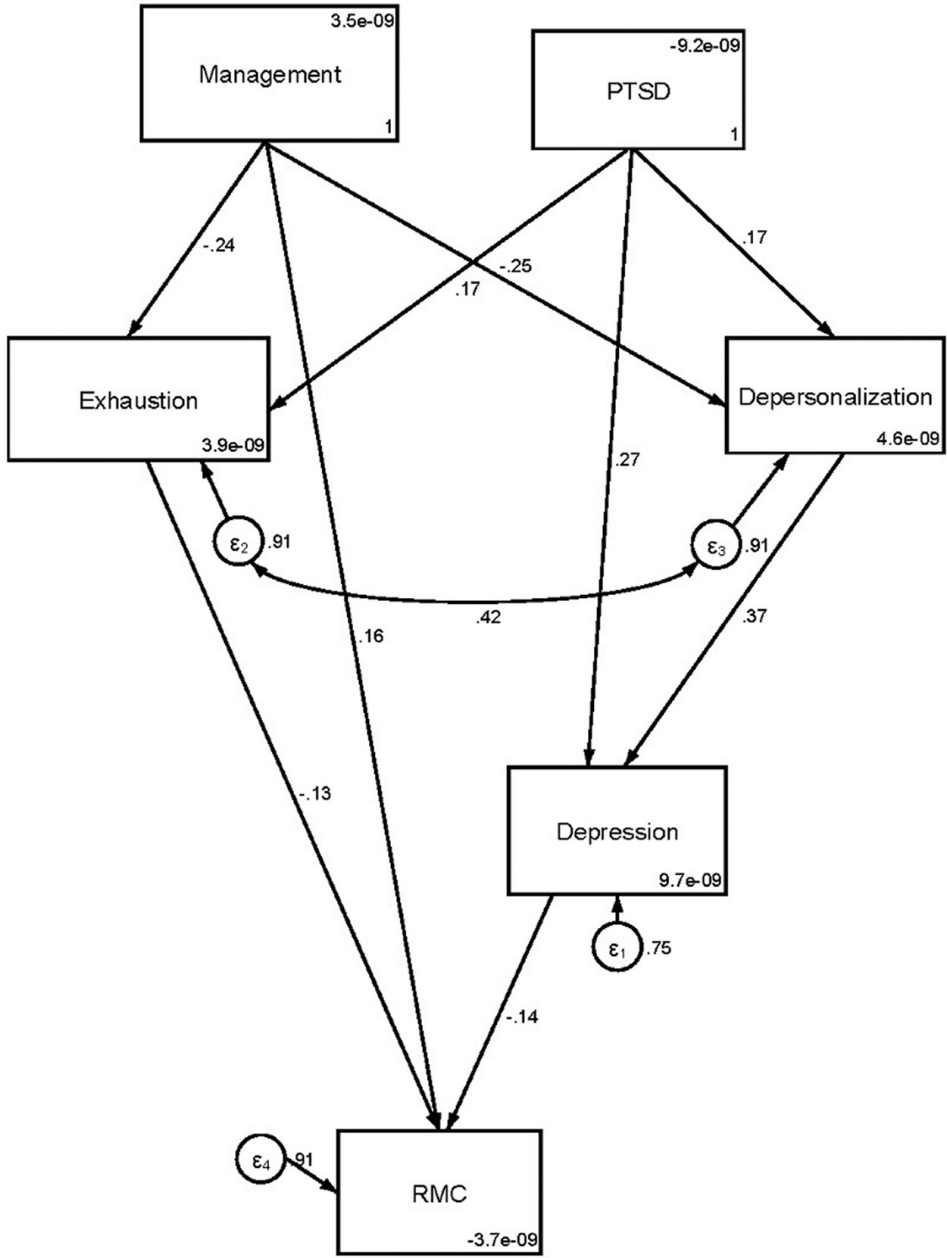
Path Diagram Depicting How Interrelated Aspects of Maternal Health Providers' Mental Health Status Might Affect Their Delivery of RMC Abbreviations: PTSD, post-traumatic stress disorder; RMC, respectful maternity care.

Finally, we used path analysis to generate a model that describes how interrelated aspects of an MH provider's mental health status might affect their delivery of RMC to women in the maternity unit. The final path diagram (Figure 3) corroborates the first-order effects observed between emotional exhaustion, depression, and management inventory that have direct relationships with RMC scores. Both emotional exhaustion and depression had significant and detrimental effects on MH providers' reports of RMC (β=−0.13 SD, *P*=.031 and β=−0.14 SD, *P*=.013, respectively), while MH providers' relationships with management had positive effects on RMC.

MH providers' relationships with management had positive effects on RMC.

The model also shows additional mediated effects on RMC. These are factors that do not directly influence RMC but are secondary correlates of depression and emotional exhaustion that may exacerbate these conditions and influence RMC indirectly. In this analysis, we found that depersonalization strongly influenced depression. For every standard deviation increase in depersonalization scores, depression increased by 0.37 SD *(P*<.001), which yields reduced RMC scores (β=−0.052, *P*=.017). PTSD had a significant association with depersonalization (β=0.17, *P*=.003), depression (β=0.27, *P*<.001) and emotional exhaustion (β=0.17, *P*=.003). A mediation analysis of these relationships suggests that PTSD has significant (β=−0.068, *P*=.002) nonspecific effects across numerous aspects of an MH provider's mental health and well-being.

Despite the detrimental effects of increased levels of PTSD, depersonalization, emotional exhaustion, and depression on RMC (using the PCMC score), we found that an MH provider's relationship with the facility management—that is, how the facility management supports their staff, whether they are perceived as making fair decisions, and how they involve MH providers in decision-making processes—can have a powerfully protective effect in this situation. Not only do positive, supportive relationships with facility management show a direct, positive association with PCMC (β=0.163, *P*=.004), but also positive relationships with facility management also appear to mitigate the effects of both depersonalization and emotional exhaustion, yielding a significant mediated effect on RMC (β=0.043, *P*=.008). We used several indices to assess goodness of fit of the path model.^34^^,^^35^ The likelihood ratio was nonsignificant (*P*=.595), root mean square of error approximation was below 0.06, standardized root mean square residual was below 0.8, and both comparative fit index and Tucker-Lewis Index were above 0.9. Thus, all relevant indices suggest close model fit. However, since cross validation was not performed with the available data, model overfitting is a possibility.

## DISCUSSION

This exploratory analysis of data from a cross-sectional survey with MH providers in Malawi examines their well-being and mental health in relation to self-reported RMC. We used the MBI, PHQ-9, and PC-PTSD scales to assess MH provider well-being, and the provider-reported PCMC scale to measure RMC. So, as was shown in the initial analysis of correlations, despite the limited explanatory power of any 1 measure, we were able to present a pathway that describes some of the complex interconnections between these factors that ultimately influences a provider's delivery of RMC.

Specifically, it appears that high scores of emotional exhaustion and depression may have a detrimental effect on MH providers' reported ability to deliver RMC. But also, while depersonalization does not directly influence RMC, it may contribute to the effects of depression or have second-order effects on RMC that are otherwise mediated through RMC. Likewise, emotional exhaustion, depersonalization, and depression may act as mediators for the effects of PTSD on RMC. If an MH provider feels supported by their supervisors (i.e., various aspects of the facility management), this has a mediating effect on both depersonalization and emotional exhaustion, as well as showing a direct, positive association with RMC.

It appears that emotional exhaustion and depression scores may have a detrimental effect on MH providers' reported ability to deliver RMC.

While it is not surprising to find that MH provider mental health influences RMC provision, there are limited studies demonstrating a clear pathway in a low-income country like Malawi. While our data are cross-sectional and vastly insufficient to indicate causality, this path model does suggest some directionality of effects within the context and confines of our dataset; we need to exercise caution in this interpretation. For instance, the relationship between depersonalization and depression may, in fact, run the opposite direction. However, in the context of the other variables included in the model, we see that depression has a direct influence on RMC, while the effects that depersonalization has on RMC are mediated through depression. Thus, despite the limitations of our data and lack of temporality, the structure of the interactions within the model suggests that burnout may be a contributing factor to depression. In fact, these findings are supported by prior longitudinal research findings from Finland, which suggest burnout may indeed be antecedent to depression.^36^

While further research is needed to improve generalizability and further develop this model of provider mental health, our findings contribute to the growing body of literature on the need to understand the situation from the health workers' viewpoints to inform policy and practice. Indeed, the idea for this analysis originated with a request from MH providers and researchers in Malawi who have long recognized the challenges faced by those working in under-resourced facilities.^8^^,^^37^

Several studies have explored health care worker behavior using a variety of scales and inventories in sub–Saharan Africa. For example, 1 study investigated the work environment that promotes motivation and performance of midlevel providers in Malawi using the MBI as well as the Healthcare Providers Work Index. Midlevel providers generally perform tasks usually associated with more highly trained workers. McAuliffe et al. found that more than 30% scored high on emotional exhaustion—similar to our findings.^38^ However, this study found higher depersonalization (17%) and lower personal accomplishment (42%) than McAuliffe et al., 5% and 27%, respectively. The Healthcare Providers Work Index described by McAuliffe has 4 subscales, and each was found to correlate with higher MBI scores.

Where the work environment was inadequately resourced, providers often felt more emotionally exhausted, and those who felt they had poor management support were more likely to seek other employment. Job satisfaction also plays an important role in provider performance.^38^ While compensation is a significant predictor of job satisfaction among providers, McAuliffe's work showed that rather, “satisfaction with current work assignments” was often the strongest correlate of overall job satisfaction. Poor relations with management and perceptions of unfair or inequitable treatment undermined job satisfaction and performance among midlevel providers in Malawi. Elsewhere it has been found that where providers have agency to make decisions, work as a team to share workload, and have a supportive management structure, they were able to overcome structural deficiencies (inadequate equipment, supplies, and space) to provide quality care.^39^

A more recent study in Kenya and Ghana found that perceived stress and burnout were critical factors associated with self-reported RMC, highlighting the need to mitigate stress among MH providers working in a challenging health system.^30^ Work conditions were also assessed, including satisfaction, perceived stress, burnout, working relationships, and hours worked. Perceived stress was measured using the 10-item Cohen perceived stress scale, and burnout was measured using the 14-item Shirom-Melamed Burnout Measure.^40^

Over the last 1 to 2 decades, work has been done on addressing barriers to quality midwifery care and MH provider stress; however, interventions on how to support providers working in challenging settings are still not widely known or used.^11^ The experiences and needs of MH providers are often overlooked, with limited understanding of how the health facility culture influences the quality of care. This includes understanding MH providers' overall well-being given their often overwhelming responsibilities in providing care in low-resource settings, with inequitable and poor remuneration and inadequate supervision.^2^^,^^5^^,^^37^^,^^38^^,^^41^^,^^42^ Our data resonate with several studies in sub-Saharan Africa, whereby providers say they are actively seeking alternative employment.^3^^,^^38^^,^^43^

From a health care provider's point of view, it is important to understand the context of their work environment and how it might affect their service delivery. The experience of MH providers, caught between an unsupportive system and dissatisfied women, has been documented as an underlying factor in the persistence of disrespectful or poor person-centered care.^4^ Health care providers often face a moral dilemma in not being able to offer quality care because of a lack of resources and limited psychological support,^11^ which also may contribute to higher stress and burnout. In a global World Health Organization consultation of midwives, a significant number of African midwives described feeling disrespected in the workplace, and 53% said they would value being listened to.^44^

Midwives need to be appropriately resourced and equipped with the right skill set and authorized with the right scope of practice.^11^^,^^43^ A study in Kenya also found that facility working relationships and environment not only impact maternity providers' emotional health (including burnout) but also can drive poor provider-client interactions and affect women's care. Moreover, the same study found that dissatisfaction with unresponsive management related to perceptions of fair job promotion led to demotivated health workers.^2^

Understanding factors that influence provider behavior (normative, structural, and behavioral) and developing pragmatic approaches to alleviate some of them may lead to improved quality of care, including more RMC. A multipronged approach is required, including empowering MH providers to deal with difficult situations and providing opportunities, such as providing group and individual counseling services, to help them overcome experiences related to high workload, trauma, or critical incidents.^41^^,^^45^^,^^46^ To be effective, interventions that are designed to promote MH provider behavior change must encourage an understanding of any entrenched attitudes, what motivates providers, values, and other more normative factors that drive how providers interact with clients, colleagues, and managers within the health system.

Understanding how mental health factors interact to influence provider behavior may aid in developing pragmatic approaches to alleviate some of these burdens, which in turn, may lead to improved quality of care.

### Limitations

Our study had several limitations. First and foremost, this analysis is exploratory and is intended as a starting point for developing testable hypotheses by illustrating a plausible network of interacting mental health and experiential factors that influence a specific behavioral outcome—the provision of RMC. While our results do indeed show that results show a possible pathway for influencing RMC, there is a wide array of potentially influential factors that were not captured in our data or included in the model, and ultimately, the validity of our findings may be limited only to the context of the data we collected.

First, data collection for this study was conducted over the telephone during an exceptional time period: the COVID-19 pandemic. While the Malawi MOH did enact supportive policies, such as permitting a companion of choice to accompany a mother to the labor and delivery and attempting to ensure health facilities were well supplied with personal protective equipment, MH providers informally described working even longer hours than usual, being short staffed as colleagues were shifted to COVID-19 response centers and being persecuted within their communities for fear of contagion. So, while conducting interviews by telephone might have altered a participant's willingness to answer uncomfortable questions, the pandemic itself certainly amplified the difficult working conditions and other stressors experienced by MH providers and presented unprecedented challenges to even the most basic interactions between medical staff and their clients.

Unfortunately, as we did not capture comparable data before the pandemic, we are unable to assess the effects of the pandemic itself on MH providers' emotional well-being. Social desirability bias related to MH providers reporting their own perceptions of RMC could underreport the issues. Additionally, no cross-validation of our final model was conducted, which increases the likelihood of model over-fit. We recommend that future research in this area employ k-fold cross-validation to refine the model and assess how it will perform with new data—or how generalizable the model is. Finally, we had insufficient sample size to include additional covariates, such as MH provider type, demographic, and other factors in the mental health pathway model.

While the generalizability of findings using a relatively small sample of MH providers from selected facilities may be limited to the Malawi context, this exploratory analysis presents a set of findings that can be further assessed in Malawi and elsewhere and contributes to a small but growing literature on measuring MH provider well-being and mental health (Kenya, Ghana, and Malawi) in relation to promoting RMC. Future research may develop a scale out of management items and use the full PTSD questionnaire, include a larger sample size, and be done in other countries to get better diversity of responses and measurement of underlying construct.

Without identifying and addressing burnout among MH providers, efforts to achieve RMC will not be reached. However, the data we have presented suggest that provider burnout and poor mental health do not occur in a vacuum. How MH providers' experiences of stress and burnout manifest may, in turn, be influenced by comorbid mental health conditions, such as PTSD or depression. For some, emotional exhaustion and depersonalization are compounded by PTSD, which further compromises their ability to deliver respectful, person-centered care. And depression, which is a diagnosable and treatable condition unto itself, provides a conduit for the deleterious influences of PTSD and depersonalization on RMC.

## CONCLUSION

If a goal for MH providers is to deliver person-centered, respectful care, then programs that support this objective must also be provider centered. It is of paramount importance to not only measure and address burnout but also address the constellation of mental health factors that influence how MH providers interact with their world. While considerable work is needed to destigmatize and expand treatment for diagnosable mental health conditions like depression and PTSD, a clear message from the data presented here is that positive, supportive relationships with facility managers that make MH providers feel seen and appreciated not only reinforce RMC but may protect against some of the more pernicious effects of burnout. Pragmatic approaches for improving teamwork, psychosocial, and managerial support for MH providers working in challenging environments may go a long way to mitigate burnout, improve MH provider well-being and, in turn, improve RMC for all women seeking MH care.

## Supplementary Material

GHSP-D-23-00008-supplements.pdf

## Notes

**Peer Reviewed**

**First published online:** August 17, 2023.

## References

[1] Bradley S. Midwives’ Perspectives on the Practice, Impact and Challenges of Delivering Respectful Maternity Care in Malawi. Dissertation. City, University of London; 2018. Accessed August 3, 2023. https://openaccess.city.ac.uk/id/eprint/20021/

[2] Ndwiga C, Warren CE, Ritter J, Sripad P, Abuya T. Exploring provider perspectives on respectful maternity care in Kenya: “Work with what you have.” Reprod Health. 2017;14(1):99. 10.1186/s12978-017-0364-8. 28830492 PMC5567891

[3] Abuya T, Warren CE, Miller N, et al. Exploring the prevalence of disrespect and abuse during childbirth in Kenya. PLoS One. 2015;10(4):e0123606. 10.1371/journal.pone.0123606. 25884566 PMC4401776

[4] Bohren MA, Vogel JP, Hunter EC, et al. The mistreatment of women during childbirth in health facilities globally: a mixed-methods systematic review. PLoS Med. 2015;12(6):e1001847. 10.1371/journal.pmed.1001847. 26126110 PMC4488322

[5] Bradley S, McCourt C, Rayment J, Parmar D. Disrespectful intrapartum care during facility-based delivery in sub-Saharan Africa: a qualitative systematic review and thematic synthesis of women’s perceptions and experiences. Soc Sci Med. 2016;169:157–170. 10.1016/j.socscimed.2016.09.039. 27723514

[6] Afulani PA, Kelly AM, Buback L, Asunka J, Kirumbi L, Lyndon A. Providers’ perceptions of disrespect and abuse during childbirth: a mixed-methods study in Kenya. Health Policy Plan. 2020;35(5):577–586. 10.1093/heapol/czaa009. 32154878 PMC7225569

[7] Mgawadere F, Unkels R, Kazembe A, van den Broek N. Factors associated with maternal mortality in Malawi: application of the three delays model. BMC Pregnancy Childbirth. 2017;17(1):219. 10.1186/s12884-017-1406-5. 28697794 PMC5506640

[8] Thorsen VC, Tharp ALT, Meguid T. High rates of burnout among maternal health staff at a referral hospital in Malawi: a cross-sectional study. BMC Nurs. 2011;10:9. 10.1186/1472-6955-10-9. 21605379 PMC3114002

[9] Flanagan SV, Razafinamanana T, Warren C, Smith J. Barriers inhibiting effective detection and management of postpartum hemorrhage during facility-based births in Madagascar: findings from a qualitative study using a behavioral science lens. BMC Pregnancy Childbirth. 2021;21(1):320. 10.1186/s12884-021-03801-w. 33888075 PMC8063356

[10] Beltman JJ, van den Akker T, Bwirire D, et al. Local health workers’ perceptions of substandard care in the management of obstetric hemorrhage in rural Malawi. BMC Pregnancy Childbirth. 2013;13:39. 10.1186/1471-2393-13-39. 23414077 PMC3583700

[11] Filby A, McConville F, Portela A. What prevents quality midwifery care? A systematic mapping of barriers in low- and middle-income countries from the provider perspective. PLoS One. 2016;11(5):e0153391. 10.1371/journal.pone.0153391. 27135248 PMC4852911

[12] Kim MH, Mazenga AC, Simon K, et al. Burnout and self-reported suboptimal patient care amongst health care workers providing HIV care in Malawi. PLoS One. 2018;13(2):e0192983. 10.1371/journal.pone.0192983. 29466443 PMC5821338

[13] Sethi R, Gupta S, Oseni L, Mtimuni A, Rashidi T, Kachale F. The prevalence of disrespect and abuse during facility-based maternity care in Malawi: evidence from direct observations of labor and delivery. Reprod Health. 2017;14(1):111. 10.1186/s12978-017-0370-x. 28877701 PMC5588731

[14] Maslach C, Jackson SE, Leiter MP. Malasch Burnout Inventory. 4th ed. Mind Garden Inc; 2018.

[15] Dugani S, Afari H, Hirschhorn LR, et al. Prevalence and factors associated with burnout among frontline primary health care providers in low- and middle-income countries: a systematic review. Gates Open Research. 2018;2:4. 10.12688/gatesopenres.12779.1. 29984356 PMC6030396

[16] Stodel JM, Stewart-Smith A. The influence of burnout on skills retention of junior doctors at Red Cross War Memorial Children’s Hospital: a case study. S Afr Med J. 2011;101(2):115–118. 10.7196/SAMJ.4431. 21678738

[17] Patel R, Bachu R, Adikey A, Malik M, Shah M. Factors related to physician burnout and its consequences: a review. Behav Sci (Basel). 2018;8(11):98. 10.3390/bs8110098. 30366419 PMC6262585

[18] Poghosyan L, Clarke SP, Finlayson M, Aiken LH. Nurse burnout and quality of care: cross-national investigation in six countries. Res Nurs Health. 2010;33(4):288–298. 10.1002/nur.20383. 20645421 PMC2908908

[19] Welp A, Meier LL, Manser T. Emotional exhaustion and workload predict clinician-rated and objective patient safety. Front Psychol. 2015;5:1573. 10.3389/fpsyg.2014.01573. 25657627 PMC4302790

[20] Hamed RA, Abd Elaziz SY, Ahmed AS. Prevalence and predictors of burnout syndrome, post-traumatic stress disorder, depression, and anxiety in nursing staff in various departments. Middle East Current Psychiatry. 2020;27(1):36. 10.1186/s43045-020-00044-x

[21] Rossouw L, Seedat S, Emsley RA, Suliman S, Hagemeister D. The prevalence of burnout and depression in medical doctors working in the Cape Town Metropolitan Municipality community healthcare clinics and district hospitals of the Provincial Government of the Western Cape: a cross-sectional study. S Afr Fam Pract. 2013;55(6):567–573. 10.1080/20786204.2013.10874418

[22] Cyr S, Marcil MJ, Marin MF, et al. Factors associated with burnout, post-traumatic stress and anxio-depressive symptoms in healthcare workers 3 months into the COVID-19 pandemic: an observational study. Front Psychiatry. 2021;12:668278. 10.3389/fpsyt.2021.668278. 34305675 PMC8295587

[23] Carmassi C, Bertelloni CA, Avella MT, et al. PTSD and burnout are related to lifetime mood spectrum in emergency healthcare operator. Clin Pract Epidemiol Ment Health. 2020;16:165–173. 10.2174/1745017902016010165. 32874191 PMC7431684

[24] Serrano-Ripoll MJ, Meneses-Echavez JF, Ricci-Cabello I, et al. Impact of viral epidemic outbreaks on mental health of healthcare workers: a rapid systematic review and meta-analysis. J Affect Disord. 2020;277:347–357. 10.1016/j.jad.2020.08.034. 32861835 PMC7443314

[25] Dewey LM, Allwood MA. When needs are high but resources are low: a study of burnout and secondary traumatic stress symptoms among nurses and nursing students in rural Uganda. Int J Stress Manag. 2022;29(1):31–43. 10.1037/str0000238

[26] Padmanabhanunni A. The cost of caring: secondary traumatic stress and burnout among lay trauma counsellors in the Western Cape Province. S Afr J Psychol. 2020;50(3):385–394. 10.1177/0081246319892898

[27] Spitzer RL, Kroenke K, Williams JB. Validation and utility of a self-report version of PRIME-MD: the PHQ primary care study. JAMA. 1999;282(18):1737–1744. 10.1001/jama.282.18.1737. 10568646

[28] Prins A, Bovin MJ, Smolenski DJ, et al. The Primary Care PTSD Screen for DSM-5 (PC-PTSD-5): development and evaluation within a veteran primary care sample. J Gen Intern Med. 2016;31(10):1206–1211. 10.1007/s11606-016-3703-5. 27170304 PMC5023594

[29] Afulani PA, Feeser K, Sudhinaraset M, Aborigo R, Montagu D, Chakraborty N. Toward the development of a short multi‐country person‐centered maternity care scale. Int J Gynaecol Obstet. 2019;146(1):80–87. 10.1002/ijgo.12827. 31004349

[30] Afulani PA, Aborigo RA, Nutor JJ, et al. Self-reported provision of person-centred maternity care among providers in Kenya and Ghana: scale validation and examination of associated factors. BMJ Glob Health. 2021;6(12):e007415. 10.1136/bmjgh-2021-007415. 34853033 PMC8638154

[31] Adewuya AO, Ola BA, Afolabi OO. Validity of the patient health questionnaire (PHQ-9) as a screening tool for depression amongst Nigerian university students. J Affect Disord. 2006;96(1-2):89–93. 10.1016/j.jad.2006.05.021. 16857265

[32] Williamson MLC, Stickley MM, Armstrong TW, Jackson K, Console K. Diagnostic accuracy of the Primary Care PTSD Screen for DSM‐5 (PC‐PTSD‐5) within a civilian primary care sample. J Clin Psychol. 2022;78(11):2299–2308. 10.1002/jclp.23405. 35763419

[33] Warren C, Njuki R, Abuya T, et al. Study protocol for promoting respectful maternity care initiative to assess, measure and design interventions to reduce disrespect and abuse during childbirth in Kenya. BMC Pregnancy Childbirth. 2013;13:21. 10.1186/1471-2393-13-21. 23347548 PMC3559298

[34] Streiner DL. Finding our way: an introduction to path analysis. Can J Psychiatry. 2005;50(2):115–122. 10.1177/070674370505000207. 15807228

[35] Hu L, Bentler PM. Cutoff criteria for fit indexes in covariance structure analysis: conventional criteria versus new alternatives. Struct Equ Modeling. 1999;6(1):1–55. 10.1080/10705519909540118

[36] Ahola K, Hakanen J. Job strain, burnout, and depressive symptoms: a prospective study among dentists. J Affect Disord. 2007;104(1-3):103–110. 10.1016/j.jad.2007.03.004. 17448543

[37] Muula AS, Maseko FC. How are health professionals earning their living in Malawi? BMC Health Serv Res. 2006;6(1):97. 10.1186/1472-6963-6-97. 16899130 PMC1555580

[38] McAuliffe E, Bowie C, Manafa O, et al. Measuring and managing the work environment of the mid-level provider – the neglected human resource. Hum Resour Health. 2009;7:13. 10.1186/1478-4491-7-13. 19228391 PMC2655277

[39] Cohen S, Kamarck T, Mermelstein R. A global measure of perceived stress. J Health Soc Behav. 1983;24(4):385–396. 10.2307/2136404. 6668417

[40] Shirom A, Melamed S. A comparison of the construct validity of two burnout measures in two groups of professionals. Int J Stress Manag. 2006;13(2):176–200. 10.1037/1072-5245.13.2.176

[41] Afulani PA, Ongeri L, Kinyua J, Temmerman M, Mendes WB, Weiss SJ. Psychological and physiological stress and burnout among maternity providers in a rural county in Kenya: individual and situational predictors. BMC Public Health. 2021;21(1):453. 10.1186/s12889-021-10453-0. 33676479 PMC7936594

[42] Zimmerman E, Caetano V, Banay R, Smith J. *Evidence Review and Analysis of Provider Behavior Change Opportunities*. Population Council; 2020. Accessed July 20, 2023. http://breakthroughactionandresearch.org/wp-content/uploads/2020/04/PBC-Lit-Review.pdf

[43] Bradley S, Kamwendo F, Chipeta E, Chimwaza W, de Pinho H, McAuliffe E. Too few staff, too many patients: a qualitative study of the impact on obstetric care providers and on quality of care in Malawi. BMC Pregnancy Childbirth. 2015;15(1):65. 10.1186/s12884-015-0492-5. 25880644 PMC4377843

[44] World Health Organization (WHO). *Midwives’ Voices, Midwives’ Realities: Findings From a Global Consultation on Providing Quality Midwifery Care*. WHO; 2016. Accessed July 20, 2023. https://apps.who.int/iris/handle/10665/250376

[45] Warren CE, Ndwiga C, Sripad P, et al. Sowing the seeds of transformative practice to actualize women’s rights to respectful maternity care: reflections from Kenya using the consolidated framework for implementation research. BMC Womens Health. 2017;17(1):69. 10.1186/s12905-017-0425-8. 28854925 PMC5577789

[46] Ndwiga C, Warren CE, Abuya T, et al. *Respectful Maternity Care Resource Package: Facilitator’s Guide*. Population Council; 2014. Accessed July 20, 2023. https://knowledgecommons.popcouncil.org/departments_sbsr-rh/1949/

