# Supplementary material for: Modeling Pathways to Describe How Maternal Health Care Providers' Mental Health Influences the Provision of Respectful Maternity Care in Malawi
Source: Glob Health Sci Pract. 2023 Nov 30;11(Suppl 1):e2300008. doi: 10.9745/GHSP-D-23-00008 (PMC10698235; doi:10.9745/GHSP-D-23-00008)
Supplement: GHSP-D-23-00008-supplements.pdf [file GHSP-D-23-00008-supplements.pdf]

Supplement to: Burnett-Zieman B, Warren CE, Chiundira F, et al. Modeling pathways to describe how maternal health providers' mental health influences the provision of respectful maternity care in Malawi. *Glob Health Sci Pract*. 2023;11(Suppl 3):e2300008.

Supplementary information questionnaires

Supplement 1: Provider-Reported Patient Centered Maternity Care

1. How often have you introduced yourself to a woman when you first see her? (e.g., "Hi, I am Anne, I will be taking care of you today")
How often have you referred to a woman by her name?(i.e., When you talk to a woman do you use her name; e.g., "Hi Mary, how are you feeling?")
How often have you explained to a woman why you are doing examinations or procedures ?(e.g., Through a vaginal exam, I will know if the cervix is opened enough for delivery.)
How often have you explained to a woman why you are giving her medicine?(e.g., I am giving you oxytocin to help you expel the placenta.)
How often have you asked a woman if she has any questions? (e.g., "Hi Mary, do you have any questions that you would like me to answer?)
How often do you allow a woman to have someone she wants, such as a family member or a friend, stay with her?
How often do you allow a woman to be in the position of their choice during the delivery?
How often do you shout at, scold, insult, threaten, or talk to a woman rudely? (Please select "yes" if you have done only one, like only shout; e.g. Can't you open your legs properly? Do not shout! Bear the pain!)
How often do you treat a woman roughly like pushing, beating, slapping, pinching, physically restraining, or covering her mouth when she is delivering at your health facility? (Please select "yes" if you have done only one.)

Supplement to: Burnett-Zieman B, Warren CE, Chiundira F, et al. Modeling pathways to describe how maternal health providers' mental health influences the provision of respectful maternity care in Malawi. *Glob Health Sci Pract*. 2023;11(Suppl 3):e2300008.

Supplement 2: Maslach Burnout Inventory

This section contains statements on JOB-RELATED FEELINGS. If you have never had this feeling, circle the "0" after the statement. Otherwise indicate how often you feel like this by selecting the response that best describes how usually feel.
1. I feel emotionally drained from my work
2. I feel used up /at the end of my patience at the end of the workday.
3. I feel fatigued or tired when I get up in the morning and have to face another day on the job.
4. I can easily understand how my patients feel about things
5. I feel I treat some patients as if they were impersonal objects
6. Working with people all day is really a strain for me.
7. I deal very effectively with the problems of my patients.
8. I feel burned-out from my work.
9. I feel I am positively influencing other people's lives.
10. I have become more callous/ hardened/insensitive toward people since I took this job.
11. I worry that this job is hardening me emotionally.
12. I feel very energetic.
13. I feel frustrated by my job.
14. I feel I am working too hard on my job.
15. I do not really care about what happens to some patients.
16. Working directly with people puts too much stress on me.
17. I can easily create a relaxed atmosphere with my patients.
18. I accomplish many worthwhile things in this job.
19. I feel exhilarated after working closely with my patients.
20. I feel like I am at the end of my rope.
21. In my work, I deal with emotional problems very calmly.
22. I feel patients blame me for some of their problems.

Supplement to: Burnett-Zieman B, Warren CE, Chiundira F, et al. Modeling pathways to describe how maternal health providers' mental health influences the provision of respectful maternity care in Malawi. *Glob Health Sci Pract*. 2023;11(Suppl 3):e2300008.

Supplement 3: Patient Health Questionnaire 9 (PHQ-9)

Over the last 2 weeks, how often have you been bothered by any of the following problems?
1. Little interest or pleasure in doing things
2. Feeling down, depressed, or hopeless
3. Trouble falling or staying asleep, or sleeping too much
4. Feeling tired or having little energy
5. Poor appetite or overeating
6. Feeling bad about yourself—or that you are a failure or have let yourself or your family down
7. Trouble concentrating on things, such as reading the newspaper or watching television
8. Moving or speaking so slowly that other people could have noticed? Or the opposite—being so fidgety or restless that you have been moving around a lot more than usual
9. Thoughts that you would be better off dead or of hurting yourself in some way

Supplement 4: Primary Care PTSD Screen for DSM-5 (PC-PTSD-5)

**Sometimes things happen to people that are unusually or especially frightening, horrible, or traumatic. For example:**<br>• a serious accident or fire<br>• a physical or sexual assault or abuse<br>• an earthquake or flood<br>• a war<br>• seeing someone be killed or seriously injured<br>• the death of a patient for whom you were providing care<br>• having a loved one die through homicide or suicide.
1. Have you ever experienced this kind of event?
**In the past month, have you… (**Select yes/no:)
2. Had nightmares about the event(s) or thought about the event(s) when you did not want to?
3. tried hard not to think about the event(s) or went out of your way to avoid situations that reminded you of the event(s)?
4. been constantly on guard, watchful, or easily startled?
5. felt numb or detached from people, activities, or your surroundings?
6. felt guilty or unable to stop blaming yourself or others for the event(s) or any problems the event(s) may have caused?
